# An Automatic Bleeding Frame and Region Detection Scheme for Wireless Capsule Endoscopy Videos Based on Interplane Intensity Variation Profile in Normalized RGB Color Space

**DOI:** 10.1155/2018/9423062

**Published:** 2018-02-25

**Authors:** Amit Kumar Kundu, Shaikh Anowarul Fattah, Mamshad Nayeem Rizve

**Affiliations:** Department of Electrical and Electronic Engineering, Bangladesh University of Engineering and Technology, Dhaka, Bangladesh

## Abstract

Wireless capsule endoscopy (WCE) is an effective video technology to diagnose gastrointestinal (GI) disease, such as bleeding. In order to avoid conventional tedious and risky manual review process of long duration WCE videos, automatic bleeding detection schemes are getting importance. In this paper, to investigate bleeding, the analysis of WCE images is carried out in normalized RGB color space as human perception of bleeding is associated with different shades of red. In the proposed method, at first, from the WCE image frame, an efficient region of interest (ROI) is extracted based on interplane intensity variation profile in normalized RGB space. Next, from the extracted ROI, the variation in the normalized green plane is presented with the help of histogram. Features are extracted from the proposed normalized green plane histograms. For classification purpose, the K-nearest neighbors classifier is employed. Moreover, bleeding zones in a bleeding image are extracted utilizing some morphological operations. For performance evaluation, 2300 WCE images obtained from 30 publicly available WCE videos are used in a tenfold cross-validation scheme and the proposed method outperforms the reported four existing methods having an accuracy of 97.86%, a sensitivity of 95.20%, and a specificity of 98.32%.

## 1. Introduction

Bleeding is a common symptom for many gastrointestinal (GI) diseases, and thus bleeding detection has great clinical importance in diagnosing relevant diseases [1]. Conventional endoscopic techniques, such as gastroscopy and colonoscopy, face problem in demonstrating small intestine and cause a lot of pain. As an alternative, wireless capsule endoscopy (WCE) is getting popularity mainly because of its noninvasive nature [2, 3]. The small-sized camera along with the transmitter provides real-time WCE video consisting numerous images. The main problem here is the long clinical review period (usually 2 hours or more) to detect bleeding in the whole GI tract [4]. Moreover, there may be only few bleeding frames or very small bleeding regions which may not be detectable by the naked eyes. Hence, automated scheme to detect bleeding has received much attention by several researchers. In this regard, the suspected blood indicator (SBI), a software delivered along with second-generation capsule, is reported to be one of the very first attempts to detect WCE bleeding images with moderate accuracy [5]. The SBI does not reduce the required interpretation time of WCE videos, which is its main goal [6]. This motivated the researchers to propose new algorithms for automatic bleeding detection in WCE videos. A region growing bleeding detection method is proposed in [7] based on some statistical features where a major limitation is the necessity of manual identification of a bleeding region at the beginning. In [8], versatile texture models are used from different color components as features, followed by an extensive feature selection scheme to detect abnormalities. In [9], a probabilistic neural network (PNN) is employed for bleeding detection based on the features extracted from pixel intensity values in two different color spaces (RGB and HSV). In order to overcome the chances of fluctuation of individual pixel intensity, ratio of pixel intensity values in different color planes is used [10]. But separable formulation to extract bleeding region from these ratios of pixel intensity values is not mentioned. Instead of pixel level operation, in [11], superpixel-based bleeding detection method is proposed, which provides significant improvement in bleeding detection performance at the expense of high computational time for feature extraction. Moreover, histogram-based features extracted from intensity distribution are widely used for bleeding detection [10, 12]. In [13], for different ranges of red values, the variation in green plane is utilized with the help of histogram for bleeding detection. Generally, in conventional methods, histograms are obtained from different color planes and cascaded to extract bleeding detection features. For example, in [14], pixel histograms obtained in *Y*, *I*, and *Q* color planes are cascaded and used as a feature. Here, due to considering the whole image, histogram-based features in cascaded form obtained from different color planes may not reflect the expected distinguishable pattern between bleeding and nonbleeding images. Also, their interpretation may become very complex. In [15], for a particular color space, three-dimensional intensities of each pixel of a WCE image are mapped into the nearest color (visual word) to obtain word-based color histogram feature. Here, one major concern is to determine the optimum number of histogram bins. Also in [15], apart from WCE image classification, bleeding region is also identified. For bleeding region detection, a two-stage saliency map is used which is extracted by mixing different color channels and using the visual contrast. Feature extraction from a WCE image, which generally consists of a large number of pixels, not only involves huge computational burden but also can degrade the classification performance, especially when only a small portion of the entire image contains bleeding. One possible solution to overcome this problem is to find a region of interest (ROI) and then extract features from that ROI. If a precise ROI is obtained and then the histogram feature is extracted from that particular ROI, a better classification can be obtained. In this case, even a single plane unlike conventional use of multiple planes may exhibit quite distinguishable characteristics, and as a result, this ROI-based feature extraction scheme not only reduces computational burden but also provides better classification. Instead of using multiple planes, it is expected that a better intensity pattern can be obtained from the histogram of a single plane. Also, obtaining feature from the histogram in a single plane inside the extracted ROI will reduce computational burden as well as computational time. In [16], an ROI-based bleeding detection scheme is proposed using statistical features extracted from a composite color space defined as *Y* · *I*/*Q*. However, a major concern here is to precisely select the ROI which directly dictates the bleeding detection performance. Hence, extraction of precise ROI in feature-based bleeding detection schemes has still a great demand. Moreover, accurate identification of bleeding zones in a bleeding image would be an added challenge.

The objective of this paper is to develop an efficient scheme for detecting bleeding images and corresponding bleeding regions based on precise ROI detection in normalized RGB color plane. Considering the interplane intensity pattern of the pixels, linearly separable criterions are proposed which can precisely detect ROI from the preprocessed WCE image. Later, histograms from pixel values of extracted ROI in normalized color planes of WCE images are analyzed and taken as features to detect bleeding frames. Histograms are obtained from pixel intensities of the *g* plane inside the corresponding ROI. After that, bin frequency values of this regional *g* plane histogram are proposed as features for bleeding detection. Finally, the K-nearest neighbor- (KNN-) supervised classifier is employed to separate bleeding and nonbleeding images. Classification performance is tested on publicly available WCE video database. Later, from the selected ROI in the detected bleeding frames, bleeding regions are extracted by using some morphological operations. Finally, a postprocessing scheme is introduced to analyze the bleeding detection performance in continuous WCE videos. In what follows, three major tasks involved in the proposed method, namely bleeding frame detection, bleeding region identification, and video data analysis, are presented in detail in three different sections followed by results and concluding remarks.

## 2. Bleeding Frame Detection

**Figure 1:**
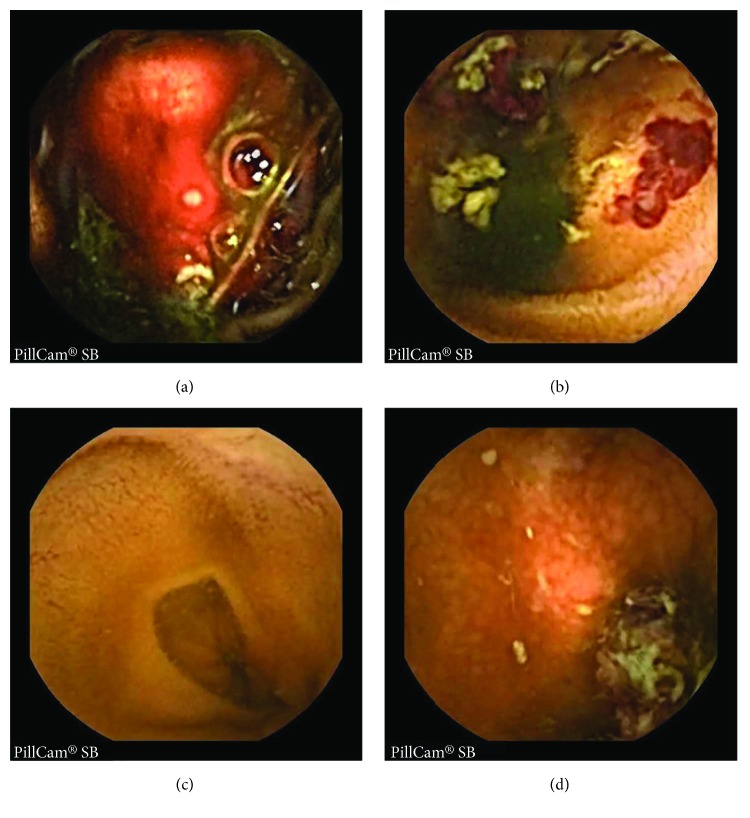
Typical WCE images; (a), (b) WCE bleeding images; (c), (d) WCE nonbleeding images.

The steps involved to detect bleeding frames from the WCE images are normalization of RGB color plane, proposed region of interest (ROI) detection, proposed feature extraction scheme, and classification with K-nearest neighbor (KNN) classifier. Typical examples of some WCE bleeding and nonbleeding images are shown in Figure 1.  Figure 1(a) represents the prominent bleeding WCE image whereas in Figure 1(b) bleeding region is very small compared to the whole image. On the other hand, Figure 1(c) represents clearly distinguishable nonbleeding images whereas Figure 1(d) represents a nonbleeding image which may have some bleeding-like pixels.

### 2.1. Normalized RGB Plane

**Figure 2:**
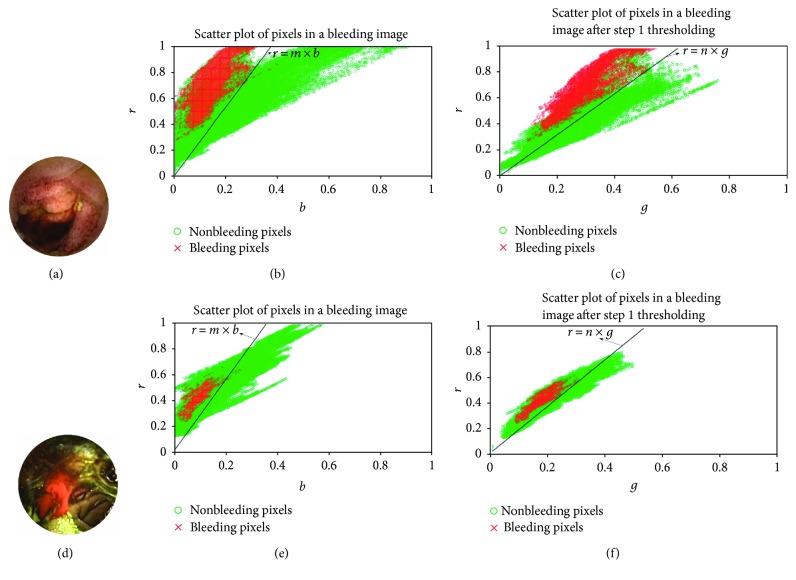
Scatter plots of normalized values; (a), (d) preprocessed bleeding images; (b), (e) *r* versus *b* intensity variation profiles in bleeding images; and (c), (f) *r* versus *g* intensity variation profiles in bleeding images after step 1 thresholding.

Generally, the central information bearing portion in a given WCE image is surrounded by some meaningless peripheral black regions as demonstrated in Figure 1(a). In order to remove these pixels, a preprocessing scheme is employed by discarding all the surrounding black pixels as demonstrated in Figures 2(a) and 2(d). A color WCE image is a snapshot of the digestive tract at a given time. However, most of the imaging devices use the RGB color space which contains both color and intensity information, that is, the RGB values are different for light red, dark red, and maroon. Therefore, it is always very difficult to recognize bleeding through an individual RGB component. Moreover, a common problem of the WCE images is that the battery of the capsule weakens over time [17]. The main disadvantage of RGB-based representation of images is that a change in the intensity leads to a change in all the three components [18]. In order to overcome this problem, in this paper, instead of RGB, normalized RGB (denoted by small alphabets *rgb*) color scheme is utilized for bleeding detection which is found less sensitive to illumination changes [19–21]. If (*i*, *j*) is the coordinate of a pixel in a WCE image, then each pixel in the WCE image is transformed to normalized RGB through the following equations:

(1) $$\begin{array}{c} r\left(i,j\right)=\frac{R\left(i,j\right)}{R\left(i,j\right)+G\left(i,j\right)+B\left(i,j\right)}, \end{array}$$

(2) $$\begin{array}{c} g\left(i,j\right)=\frac{G\left(i,j\right)}{R\left(i,j\right)+G\left(i,j\right)+B\left(i,j\right)}, \end{array}$$

(3) $$\begin{array}{c} b\left(i,j\right)=\frac{B\left(i,j\right)}{R\left(i,j\right)+G\left(i,j\right)+B\left(i,j\right)}. \end{array}$$

Since the range *R*(*i*, *j*), *G*(*i*, *j*), or *B*(*i*, *j*) is from 0 to 255, the value of *r*(*i*, *j*), *g*(*i*, *j*), and *b*(*i*, *j*) can vary from 0 to 1.

### 2.2. Proposed Region of Interest Detection

**Figure 3:**
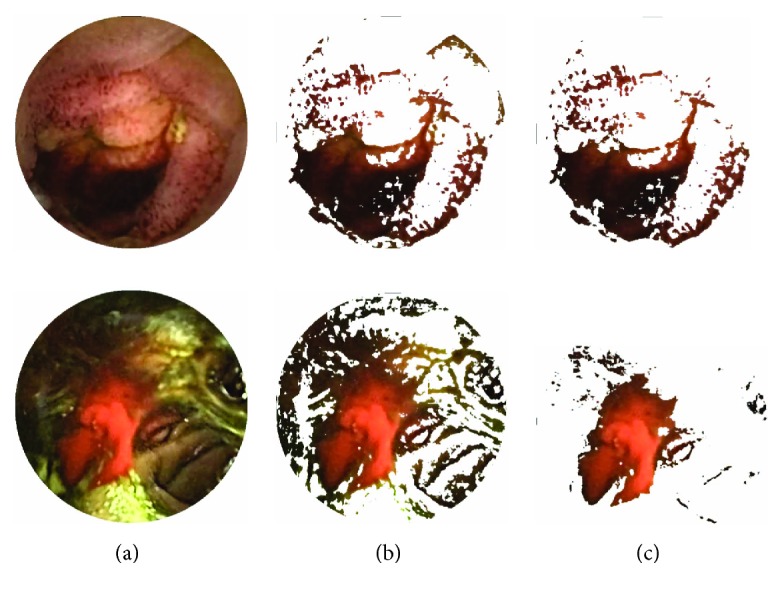
ROI extraction steps; (a) preprocessed bleeding images; (b) extracted image after first step thresholding; and (c) extracted ROI.

In order to classify bleeding and nonbleeding images, descriptive features from a given WCE image need to be extracted. In most of the bleeding detection schemes, features are extracted from the entire WCE image considering all pixels [4, 11]. Bleeding region in a WCE image, in comparison to the whole area, is generally observed in a smaller zone no matter how scattered or concentrated. Such a bleeding region is generally absent in nonbleeding images. Hence, instead of considering the whole image, if a certain smaller region is considered, more discriminating features are expected to be obtained from bleeding and nonbleeding images. In [22], a region of interest segmentation scheme is implemented as a preprocessing step to discard the meaningless areas found in the initial capsule endoscopy images. Considering the entire image for feature extraction not only increases computational burden but also increases the amount of undesired information. For example, in some bleeding images, there may exist a large nonbleeding region. In this case, if features are extracted by considering all pixels of the entire image, extracted features representing the bleeding image may be highly contaminated due to the dominance of nonbleeding pixels. In order to overcome this problem, in the proposed method, first a region of interest (ROI) is identified and then that ROI is used for feature extraction followed by bleeding image classification. Twofold advantages in ROI-based feature extraction are reduction in computational burden and enhancement in the quality of extracted features. In order to find a simple and efficient ROI detection scheme, pixel intensities in normalized plane (*rgb*) of several WCE bleeding images are analyzed. To demonstrate the distribution of normalized pixel intensities in preprocessed bleeding images, a scatter plot is shown in Figure 2. In Figures 2(a) and 2(d), two preprocessed bleeding images are demonstrated. The distribution of *r* and *b* values of bleeding and nonbleeding pixels of these preprocessed images is demonstrated in Figures 2(b) and 2(e), respectively. The bleeding and nonbleeding pixels are recognized by comparing the images with their corresponding ground truth images marked by the expert clinicians. From these figures, it is evident that most of the nonbleeding pixels are quite distinctive from the bleeding pixels in *r* − *b* 2D plane. For obtaining a precise separation between bleeding and nonbleeding pixels in *r* − *b* plane, although choice of a nonlinear separating boundary is more appropriate, a simple linear separating boundary may also be taken into consideration which is much easier to handle. Therefore, a simple linearly separable condition is proposed as

(4) $$\begin{array}{c} r\left(i,j\right)\geq m\times b\left(i,j\right), \end{array}$$

where (*i*, *j*) is the coordinate of a pixel in a WCE image and *m* is the slope of the discriminating straight line in *r* − *b* plane. The pixels in a preprocessed image that do not follow condition (4) are discarded. It can be observed that due to linear approximation for nonlinearly separated zones in *r* − *b* plane, some additional pixels apart from the desired pixels still remain in the selected pixels. For further analysis, the distribution of *r* and *g* intensities of the remaining pixels is shown in Figures 2(c) and 2(f), respectively. Again, it can be observed that further separation is possible in *r* − *g* domain similar to *r* − *b* plane, a separation that can be approximated to discard the remaining nonbleeding pixels. Therefore, a second step condition is proposed as

(5) $$\begin{array}{c} r\left(i,j\right)\geq n\times g\left(i,j\right), \end{array}$$

where *n* is the slope of the discriminating straight line in *r* − *g* plane. Out of the remaining pixels after first step thresholding, those pixels are discarded that do not follow condition (5). The remaining pixels are considered as the desired ROI in a WCE image. As the desired ROI can be extracted by imposing two linearly separable conditions after analyzing interplane intensity variation profiles and these conditions incorporate all three *r*, *g*, and *b* pixel intensities, therefore, it is redundant to consider *g* − *b* plane. In order to find appropriate values of *m* and *n*, first, wide ranges of *m* and *n* values are tested on several bleeding images by comparing them with their corresponding ground truth images marked by expert clinicians.  Figure 3 illustrates the ROI detection procedure. In Figure 3(a), a preprocessed WCE image is shown.  Figure 3(b) shows the pixels that follow condition (4) with a suitable value of *m*.  Figure 3(c) shows the pixels that remain in the ROI. These pixels are chosen out of the remaining pixels from Figure 3(b) that follow condition (5) with a suitable value of *n*.

**Figure 4:**
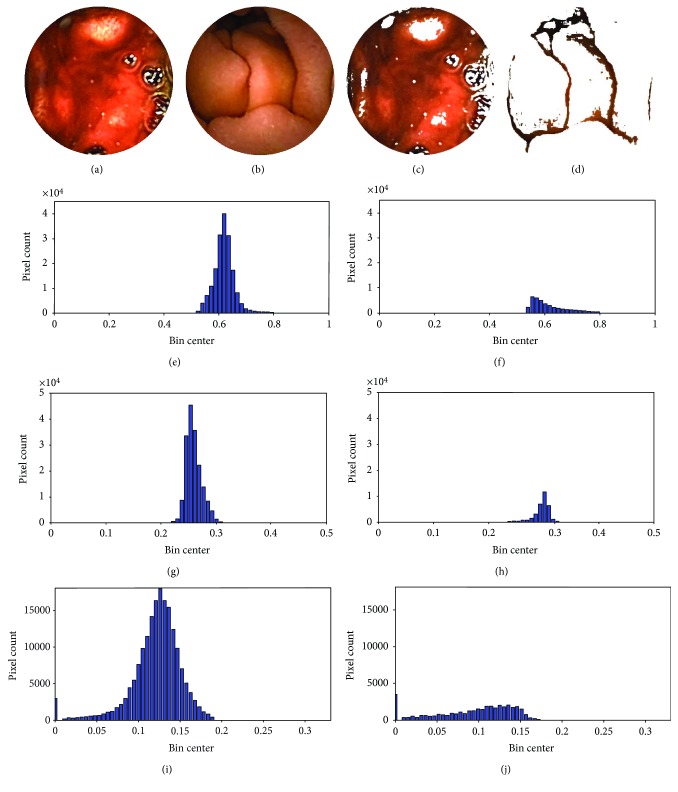
Histogram of ROI in normalized plane; (a) preprocessed bleeding image; (b) preprocessed nonbleeding image; (c) ROI of a bleeding image; (d) ROI of a nonbleeding image; (e) distribution of *r* values inside the ROI of a bleeding image; (f) distribution of *r* values inside the ROI of a nonbleeding image; (g) distribution of *g* values inside the ROI of a bleeding image; (h) distribution of *g* values inside the ROI of a nonbleeding image; (i) distribution of *b* values inside the ROI of a bleeding image; (j) distribution of *b* values inside the ROI of a nonbleeding image.

ROI segmentation provides a probable region of bleeding in a test image which does not guarantee whether the test image is a bleeding image or a nonbleeding image. Even for a nonbleeding image, an ROI will be extracted, may be small or scattered, because of the presence of bleeding-like pixels (mentioned in Figure 4(d)). As a result, to obtain a decision on the class label of a test image, a classification step is required. In fact, the idea of extracting ROI is to assist the feature-based classification scheme developed in the proposed method, not to take a final decision just looking at the presence or absence of ROI. It will only help to reduce the search space for feature extraction and also provide better accuracy. Therefore, in order to support the claim whether an image is bleeding or not, features must be extracted only on the extracted ROI for classification.

### 2.3. Proposed Feature Extraction Scheme

For any pattern recognition problem, feature extraction is the most challenging task. Therefore, the performance of a bleeding detection scheme like any pattern recognition problem highly depends on quality of the extracted features. However, one of the major obstacles in extraction of quality features is that the bleeding zone in a bleeding frame may attain any arbitrary shape covering very small to very large areas. This random nature of bleeding zones creates problem when overall statistics of the entire image like mean pixel value and minimum, maximum, or median values are used as features when ROI is not available, therefore, often results in contamination of extracted features where small bleeding zones are surrounded by large nonbleeding zones. However, when the extracted ROI is available, one may consider that feature normalization with respect to the target area can overcome the problem of varying sizes of the region under feature extraction. But, the major concern here is the number and the choice of statistical features required for better discrimination between bleeding and nonbleeding images. In order to overcome these problems, it is to be ensured that the characteristics of the bleeding zone, no matter how small or large, must contribute independently in the feature vector. Hence, in this paper, histogram of normalized planes at the ROI is proposed as features. Histogram-based features can clearly reflect the bleeding areas no matter how small or large in certain bins, and the property of bleeding is also preserved. In the proposed method, all the pixels of ROI extracted from a normalized plane of a WCE image are taken to perform histogram and frequency in each bin is measured. Therefore, this histogram-based representation of image pixels should ensure the presence of any group of bleeding pixels, no matter how small it is, independently in the feature vector. As this type of representation reflects the presence of any group of bleeding pixels in the feature vector, therefore, it is better to utilize histogram-based feature than to take only the total number of pixels inside the ROI as feature. As bleeding is determined through human perception of colors, it is expected that the histogram representation of bleeding and nonbleeding images should differ significantly and ensure the extraction of quality features. To demonstrate the difference between pixel histograms of bleeding and nonbleeding images, 64 bin histograms of selected ROI in normalized color planes are shown in Figure 4. The preprocessed sample bleeding and nonbleeding images are presented in Figures 4(a) and 4(b), respectively. Figures 4(c) and 4(d) represent the extracted ROI of bleeding and nonbleeding images. The histogram values of extracted ROI for both bleeding and nonbleeding images in *r* plane are shown in Figures 4(e) and 4(f), respectively. It can be seen that, though both the bleeding and nonbleeding histograms exhibit similar distributions, the pixel count differs significantly; this corresponds to extraction of highly distinguishable feature. In Figures 4(g)–4(j), the histogram values of extracted ROI for both bleeding and nonbleeding images in *g* plane and in *b* plane are shown, respectively. Similar type of characteristics can be observed in case of *g* plane and *b* plane. Finally, in order to acquire the final feature vector, bin frequencies of histogram at the extracted ROI in discriminating color plane are used.

### 2.4. K-Nearest Neighbor (KNN) Classifier

After the extraction of quality features, the widely used K-nearest neighbor- (KNN-) supervised classifier is used for classifying the bleeding and the nonbleeding images. In the K-nearest neighbor classifier, a distance function is computed between the train and test data sets. Then distances from K neighboring train data sets are considered to classify a test data of WCE image frame. A class membership is given to an image frame by the KNN classifier after classification. The class label assigned to a test object is determined from the votes of the majority *K* nearest neighbors. In the proposed method, to classify a test data, the Euclidean distance is used for considering the class labels of K nearest image patterns. After extensive experimentation with different values of *K*, a suitable value of *K* is determined to attain the best output.

## 3. Bleeding Region Detection

Once a bleeding image is detected successfully, automatic marking of the bleeding region will be initially helpful for the physicians to analyze the diseases. Automatic bleeding region detection can provide several benefits, such as quick visualization of the bleeding regions and exploring the changes in bleeding characteristics in consecutive video frames. Therefore, a bleeding region detection scheme is described in this section.

### 3.1. ROI Extraction

After obtaining the bleeding image frames, the bleeding region is detected in those bleeding image frames to be discussed in this section. Analyzing different WCE bleeding images, it is found that for WCE bleeding images the proposed two-step ROI extraction algorithm is sufficient to detect the bleeding region in those images. At first, a preprocessed bleeding WCE image is taken and ROI is extracted using the conditions (4) and (5) as shown in Figure 3. An ROI appears clearly like a mask, which contains possible bleeding region in a bleeding image.

### 3.2. Morphological Operations

Extraction of ROI provides a set of probable bleeding pixels in a bleeding image. Generally, a single isolated pixel may not be a candidate for the bleeding zone. Such pixels may arise due to intensity variation. Moreover, homogeneity is also an important issue to detect a smooth bleeding region in a bleeding image. In the proposed bleeding region detection method, two stage morphological operations are carried out on a bleeding image [23]. At first, the morphological erosion is done to remove small-scale details from a binary image which simultaneously reduces the size of regions of interest, too. This removal of small-scale details reduces the discretely located bleeding pixels in the out image. After that, morphological dilation is done to join disparate elements in an image. Thus, the morphological dilation makes a previously discontinuous bleeding region continuous.

## 4. Bleeding Video Analysis

### 4.1. Postprocessing

After bleeding image detection, the proposed scheme is applied to different continuous bleeding videos. As a nonbleeding frame in a bleeding video cannot occur in a discontinuous manner, a postprocessing scheme is proposed in video analysis to remove this discontinuity. If a frame is detected as nonbleeding, whereas the previous and the next image frames are recognized as bleeding ones, the frame is declared to be a bleeding one. This postprocessing scheme helps to remove the discontinuity in occurrence of nonbleeding frames in a continuous video and thus to remove falsely detected images.

## 5. Result and Discussion

In this section, the experimental results obtained from the proposed method are presented to show and compare the efficiency of the proposed method by considering 2300 WCE images. These WCE images are selected from publicly available and very widely used 30 WCE videos [24]. Among these 2300 WCE images, 450 show signs of bleeding and the rest of them show signs of nonbleeding.

### 5.1. Parameter Identification

65 bleeding images are used to determine the appropriate values of *m* and *n* by comparing them with their corresponding ground truth images marked by expert clinicians. At first, each of these 65 images is used to check pixel level accuracy of step 1 thresholding. This pixel level accuracy can be defined as

(6) $$\begin{array}{c} {Accuracy}_{pixel}=\frac{N_{tbp}+N_{tnbp}}{N_{t}}, \end{array}$$

**Figure 5:**
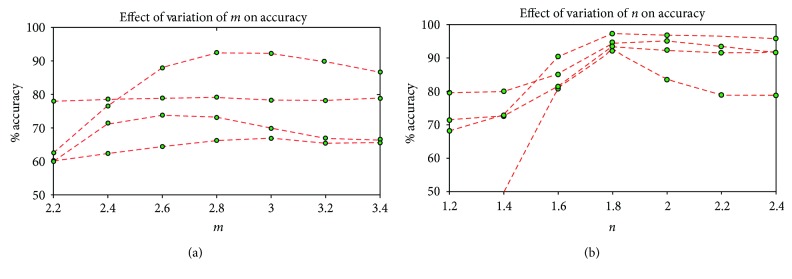
Effect of *m* and *n* on pixel level accuracy considering 4 images; (a) accuracy of step 1 thresholding using various *m*; (b) accuracy of extracted ROI using various *n*.

**Figure 6:**
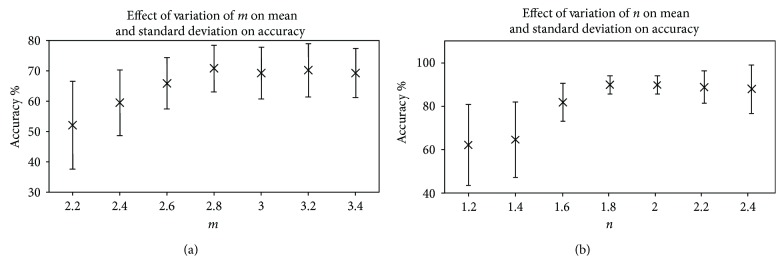
Effect of *m* and *n* on pixel level accuracy considering 65 images; (a) mean and standard deviation of accuracy of step 1 thresholding using various *m*; (b) mean and standard deviation of accuracy of extracted ROI using various *n*.

Here, *N*_tbp_, *N*_tnbp_, and *N*_t_ are the number of true bleeding pixels, the number of true nonbleeding, and the total number of pixels in a preprocessed WCE image, respectively. For demonstration purpose, the pixel level accuracy of step 1 thresholding of 4 images on varied ranges of *m* values is shown in Figure 5(a). It is evident from analysis that *m* = 2.8 has the highest pixel level accuracy percentage for most of the images. In Figure 6(a), mean and standard deviation of pixel level accuracy of step 1 thresholding are demonstrated using different values of *m* for 65 bleeding images. From the statistical measures, it is observed that *m* = 2.8 provides the highest accuracy mean (70.71%) and the lowest standard deviation (7.63%). Next, to find the appropriate value of *n*, wide ranges of *n* values are tested on those 65 bleeding images to check ROI detection accuracy keeping *m* fixed to 2.8. To demonstrate, the pixel level accuracy of ROI segmentation of 4 images on varied ranges of *n* values while keeping *m* to 2.8 is shown in Figure 5(b). It is clear from the figure that both *n* = 1.8 and 2.0 have almost the highest equal accuracy percentage for *m* = 2.8. In Figure 6(b), mean and standard deviation of pixel level accuracy of ROI detection are demonstrated using different values of *n* keeping *m* = 2.8 for 65 bleeding images. It is evident from the statistical measures that ROI segmentation with *m* = 2.8 and *n* = 2.0 provides the highest pixel level mean accuracy (88.54%) and the lowest standard deviation (3.64%). However, one may think to find the appropriate values of *m* and *n* simultaneously. But if the appropriate values of *m* and *n* are searched sequentially, then the search space to find the appropriate values of *m* and *n* will definitely reduce.

### 5.2. Performance of Bleeding Frame Detection

The initial size of these images is 576 × 576 pixels. After removing the square-shaped peripheral black region, the images become size 512 × 512 pixels. Then, the corner black pixels are removed to obtain the circular information bearing region. After that, each image is converted to *rgb* color space from RGB color space using the relationships provided in (1), (2), and (3), respectively. Then, the ROI is extracted from each bleeding and nonbleeding image using the relationships provided in (4) and (5). Histograms are obtained individually from *g* and *b* planes for each image at the corresponding ROI keeping the number of bins constant for every image. The bin centers are computed by equally dividing the overall range of *g* and *b* values into the number of bins chosen. Finally, by taking the frequency at each bin of the histogram inside the ROI provides the proposed feature vector. A KNN classifier is used for classification. Tenfold cross-validation scheme is used to evaluate the classification performance.

During classification of bleeding and nonbleeding images, four distinct cases may arise regarding the detection of WCE bleeding images, namely, false bleeding detection (F_b_), false nonbleeding detection (F_nb_), true bleeding detection (T_b_), and true nonbleeding detection (T_nb_). To evaluate the performance of the bleeding detection method, sensitivity, specificity, and accuracy [25] are ideal criterions which can be calculated as follows:

(7) $$\begin{array}{c} Sensitivity=\frac{\sum T_{b}}{\sum T_{b}+\sum F_{nb}}, \end{array}$$

(8) $$\begin{array}{c} Specificity=\frac{\sum T_{nb}}{\sum T_{nb}+\sum F_{b}}, \end{array}$$

(9) $$\begin{array}{c} Accuracy=\frac{\sum T_{b}+\sum T_{nb}}{\sum T_{b}+\sum F_{nb}+\sum T_{nb}+\sum F_{b}}. \end{array}$$

**Figure 7:**
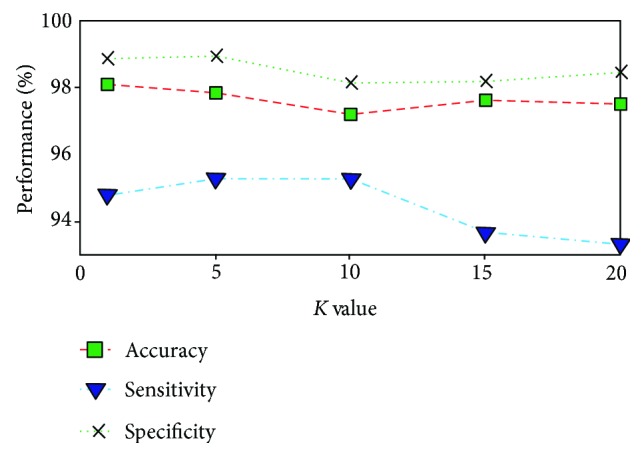
Effect of variation of *K* of KNN classifier on classification performance.

**Figure 8:**
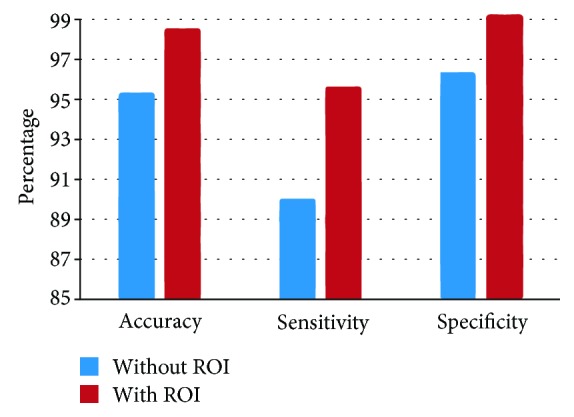
Performance comparison between normal and segmented images in *g* plane.

To extract features from histograms, different numbers of bins are used in the proposed method. The results are shown in Table 1. These features are used to investigate the bleeding detection efficiency of the proposed method. Considering the size of the feature vector, the 64 bin histograms of *g* plane for selected ROI show the best result among them with an accuracy of 97.86%, sensitivity of 95.20%, and specificity of 98.32%. In a similar way, different values of *K* are used in the KNN classifier to evaluate the performance of the proposed method. *K* = 5 shows the best result among them. The results are shown in Figure 7. In these cases, the appropriate number of bins and the value of *K* are determined globally. However, one may consider to choose histogram statistics instead of histogram bin frequencies as features. To demonstrate the goodness of histogram statistics, different statistical measures, such as mean, variance, skewness, kurtosis, and energy of *g* plane histogram are taken as features in cascade. The classification performance of histogram statistics is evaluated using KNN classifier with *K* = 5. But these features show poor performance having an accuracy of 86.79%, a sensitivity of 57.89%, and a specificity of 93.52%. Therefore, it is evident from the classification performance of histogram statistics that it is not a good discriminating feature. In Table 2, comparison among results obtained from 64 bin histograms of different color planes such as *r*, *g*, and *b* planes of normalized RGB color domain, *H* plane of *HSV* color domain, *Y* and *Q* planes of *YIQ* color domain, and *L*, *A*, and *B* planes of *CIE* − *LAB* color domain using the same ROI and the same KNN classifier with *K* = 5 is demonstrated. Similarly, in Figure 8, comparison between results obtained in *g* plane with ROI and the whole image is shown. From Figure 8 and Table 2, it is evident that the *g* plane histogram using ROI shows the best result in terms of all three performance indices. Finally, to assess the result obtained by the proposed method, it is compared with those obtained by the methods proposed in [12, 16, 26] and the uniform local binary pattern (LBP) feature proposed in [4]; here, the LBP features are extracted from RGB color plane. In [12], RGB-indexed image is used for feature extraction which requires binary coding for each image. In [16], a transformed color plane *Y* · *I*/*Q* is proposed but this method uses hard *Q* = 0 thresholding. For consistency of comparison, KNN classifier with the same value of *K* (*K* = 5) is used in all four methods. The results are demonstrated in Table 3. From the data represented in Table 3, it is evident that the proposed method performs better than the other four methods in terms of all three performance indices. Among these performance indices, sensitivity is the most important one as it represents the true bleeding detection accuracy and the proposed method outperforms the other four methods in terms of sensitivity with a significant margin.

### 5.3. Performance of Bleeding Region Detection

After obtaining the bleeding frames, experimental results obtained from the bleeding region detection are mentioned in this section. The efficiency of the bleeding region detection scheme is presented by considering 65 WCE bleeding images. The preprocessed images are converted to *rgb* color space from RGB color space using the relationships provided in (1), (2), and (3), respectively. Then, the ROI is extracted from the bleeding images using the conditions in (4) and (5). After that, two stage morphological operations are carried out on a bleeding image. At first, the morphological erosion is done to remove small-scale details from a binary image followed by morphological dilation to join disparate elements in an image. To quantitatively assess the region detection performance, a pixel-based comparison between the bleeding regions and the ground truth labeled by the expert clinicians is performed. The three performance indices used in this experiment are the false negative ratio (FNR), the false positive ratio (FPR), and precision [27, 28] which are calculated as

(10) $$\begin{array}{c} FNR=\frac{\sum F_{N}}{\sum F_{N}+\sum T_{P}}, \end{array}$$

(11) $$\begin{array}{c} FPR=\frac{\sum F_{P}}{\sum F_{P}+\sum T_{N}}, \end{array}$$

(12) $$\begin{array}{c} Precision=\frac{\sum T_{P}}{\sum T_{P}+\sum F_{P}}. \end{array}$$

**Figure 9:**
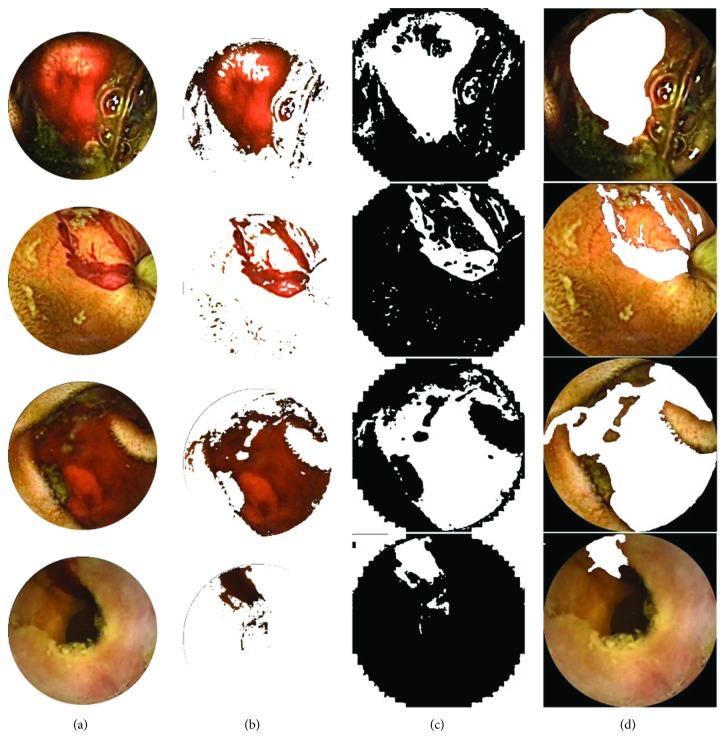
Examples of bleeding region detection; (a) preprocessed bleeding image; (b) extracted ROI; (c) output bleeding region; and (d) ground truth.

Here, true positive (T_P_) cases are when the bleeding pixels are correctly labeled as bleeding while false positive (F_P_) are the ones incorrectly labeled as the bleeding. True negative (T_N_) represent the regions that are correctly labeled as nonbleeding while the false negative (F_N_) represent the regions which are not labeled as the bleeding but should have been. In Figure 9, qualitative results for the bleeding area detection based on the extracted ROI are presented using four different preprocessed bleeding images. The first column is the preprocessed bleeding images while the second column shows extracted ROI of corresponding images, and the third column shows the detected bleeding region after morphological operations. The final column represents the ground truth for the bleeding area labeled by the expert clinicians. It can be observed that the bleeding areas are accurately detected by the proposed bleeding region detection scheme. To assess the result obtained by the proposed bleeding region detection scheme, it is compared with those obtained by the method proposed in [15]. A two-stage saliency extraction method to localize the bleeding areas in WCE images is proposed in [15]. The results are demonstrated in Table 4. From the data represented in Table 4, it is evident that the proposed method performs better than the method in [15] in terms of precision and FPR.

### 5.4. Performance in Continuous Bleeding Videos

In this subsection, the experimental results obtained from the proposed method are presented to show the efficiency of the proposed method by considering five continuous videos. Each video is one-minute long and has 100 image frames. Each image frame in those five videos is classified according to the proposed bleeding frame detection scheme. Then, the postprocessing scheme is followed to remove the discontinuity in occurrence of nonbleeding frames in a continuous video. To evaluate the performance, sensitivity, specificity, and accuracy are used as criterions. Leave-one-out cross validation scheme is used to evaluate the classification performance. The results are demonstrated in Table 5. This is to be noted that in video number 5 all the images are bleeding images. Therefore, the term specificity is not defined for that video. The excellent result in the table ensures the goodness of the proposed segmented image-based histogram feature.

## 6. Conclusion

In this paper, an efficient ROI extraction scheme is proposed based on *rgb* domain in a WCE image. Histogram representation of a WCE image in the extracted ROI in normalized plane is found very suitable for discriminating bleeding and nonbleeding images. It is observed that the use of *rgb* domain histogram provides significantly better performance than that of conventional RGB color space. Especially the bin frequencies of the *rgb* histogram inside the ROI, the difference of the number of pixels in two cases (bleeding and nonbleeding) is found very prominent. Therefore, all bin frequencies are used in the proposed feature vector. Moreover, it is observed that the use of histogram in *g* plane provides superior performance in comparison to *r* or *b* plane. For the purpose of classification, the simplest KNN classifier is employed which offers ease of implementation. The performance of proposed features in classifying bleeding and nonbleeding images is evaluated in terms of accuracy, specificity, and sensitivity, and it turns out that the proposed method outperforms other four compared methods in terms of all three performance indices. Out of these detected bleeding images, bleeding region is detected based on the extracted ROI. Morphological operations in region detection helps to make the bleeding region smooth. Finally, the proposed bleeding detection method is applied to several continuous videos. The performance of the method in continuous videos ensures the goodness of the feature. Therefore, the proposed automatic bleeding image detection scheme with improved performance will reduce the burden of the clinicians in reviewing large number of WCE images.

## Figures and Tables

**Table 1:** Effect variation of histogram bin numbers on classification performance of the proposed method.

Histogram bin	Accuracy	Sensitivity	Specificity
16 bin	96.82%	90.97%	97.89%
32 bin	97.37%	94.66%	98.01%
64 bin	**97.86%**	**95.20%**	**98.32%**
128 bin	97.20%	94.96%	97.97%

**Table 2:** Performance variation of different color domains.

Color planes	Accuracy	Sensitivity	Specificity
*r*	94.81%	84.02%	97.36%
*g*	**97.86%**	**95.20%**	**98.32%**
*b*	96.23%	91.67%	97.29%
H	97.15%	93.64%	97.99%
Y	95.56%	92.84%	96.17%
Q	96.82%	92.66%	97.70%
L	94.91%	82.43%	97.81%
A	95.67%	85.87%	97.95%
B	95.87%	87.63%	97.80%

**Table 3:** Comparison of classification accuracy among different methods (%).

Parameter	Uniform LBP [4]	Method in [26]	Method in [12]	Method in [16]	Proposed method
Accuracy	91.50	77.15	94.50	93.00	**97.86**
Sensitivity	79.25	83.50	93.00	93.50	**95.20**
Specificity	94.56	75.69	94.88	94.00	**98.32**

**Table 4:** Performance of bleeding region detection.

Method	Precision	FPR	FNR
Proposed method	**88.38%**	**6.78%**	**33.13%**
Method in [15]	85.03%	7.68%	38.13%

**Table 5:** Performance in continuous videos.

Video number	Accuracy	Sensitivity	Specificity
1	87.0%	95.5%	70%
2	96.0%	80.0%	97.0%
3	85.0%	87.5%	85.0%
4	91.0%	79.%	96.0%
5	87.0%	87.0%	—
